# Free breathing contrast-enhanced time-resolved magnetic resonance angiography in congenital heart disease

**DOI:** 10.1186/1532-429X-17-S1-O65

**Published:** 2015-02-03

**Authors:** Jennifer A Steeden, Bejal Pandya, Oliver Tann, Vivek Muthurangu

**Affiliations:** 1 UCL Centre for Cardiovascular Imaging, London, UK; 2 Cardiorespiratory Unit, Great Ormond Street Hospital for Children, London, UK; 3 University College London, The Heart Hospital, London, UK

## Background

Contrast enhanced magnetic resonance angiography (MRA) is generally performed during a long breath-hold (BH), limiting its use in infants and children. Time resolved MR angiography (TRA) often use data sharing techniques, making it is necessary to acquire data during a BH. This study proposes a high-resolution free breathing (FB) TRA sequence for use in adults and children with congenital heart disease (CHD).

## Methods

A TRA sequence was developed by combining spiral trajectories with sensitivity encoding (SENSE, x4 *kx-ky* and x2 *kz*) and partial Fourier (75% *kz*). Total acceleration was 10.7x and enabled acquisition of a high resolution volume (2.3x2.3x2.3mm) every 1.3s with no data sharing. Importantly, this allowed data to be acquired during FB. Conventional BH-MRA and FB-TRA data was acquired in 45 patients with CHD.

Diagnostic accuracy was assessed by 2 evaluators. The diagnosis were compared to those in the clinical CMR report (assessed from the whole CMR examination, including 3D whole heart and selected cine, black blood and flow imaging).

Vessel diameter measurements were made in all patients, at 6 positions; sinotubular junction (Ao1), proximal descending aorta (Ao2), descending aorta at the diaphragm (Ao3), main pulmonary artery (MPA), mid left pulmonary artery (LPA), and mid right pulmonary artery (RPA).

Additionally, we calculated quantitative image quality for both sequences at Ao1 and MPA, using measures of signal-to-noise ratio (SNR), contrast-to-noise ratio (CNR), relative contrast (RC) and edge sharpness.

## Results

The median age of the patients was 23.1±15.7years (range: 8-80 years, 13 of whom were <18 years old). FB-TRA data was successfully acquired using in all 45 patients.

BH-MRA provided overall diagnostic accuracy of 82%, and FB-TRA of 87%, with no statistical difference between the 2 sequences (P=0.77). The specific ability of the sequences to assess stenosis (N=13) found sensitivities of 62% and 69% for BH-MRA and FB-TRA, respectively (P=0.56), with both having a specificity of 100%. The specific ability of the sequences to assess dilation (N=53) found sensitivities of 98% and 100% for BH-MRA and FB-TRA, respectively (P=0.32), with both having a specificity of 100%.

Vessel diameters measurements (table 1) made in 234 vessel segments showed excellent agreement between the 2 techniques (r=0.98, P<0.05), with no bias (0.0mm,P=0.71), and clinically acceptable limits of agreement (-2.7 to +2.8mm).

**Table 1:** Vessel diameter measurements from the two angiography sequences

Vessel	N	BH-MRA (mm)	FB-TRA (mm)	Bias* (mm)	Limits of agreement* (mm)	Correlation coefficient* (r)	P-value*
Ao1	43	29 ± 6 (range: 21 to 46)	29 ± 6 (range: 19 to 47)	-0.2	-2.7 to 2.3	0.98	P=0.40

Ao2	43	21 ± 5 (range: 14 to 38)	21 ± 6 (range: 14 to 40)	0.4	-3.0 to 3.7	0.96	P=0.18

Ao3	43	19 ± 4 (range: 13 to 32)	19 ± 4 (range: 14 to 34)	0.4	-2.0 to 2.7	0.96	P=0.06

MPA	35	29 ± 7 (range: 17 to 47)	29 ± 7 (range: 16 to 46)	0.1	-2.9 to 3.0	0.98	P=0.82

RPA	35	22 ± 6 (range: 13 to 35)	21 ± 5 (range: 13 to 34)	-0.3	-3.1 to 2.5	0.97	P=0.24

LPA	35	20 ± 5 (range: 8 to 31)	20 ± 5 (range: 8 to 31)	-0.3	-2.5 to 2.0	0.98	P=0.14

Total	234	23 ± 7 (range: 8 to 47)	23 ± 7 (range: 8 to 47)	0.0	-2.7 to 2.8	0.98	P=0.71

* Calculated with BH-MRA sequence

**Figure 1:**
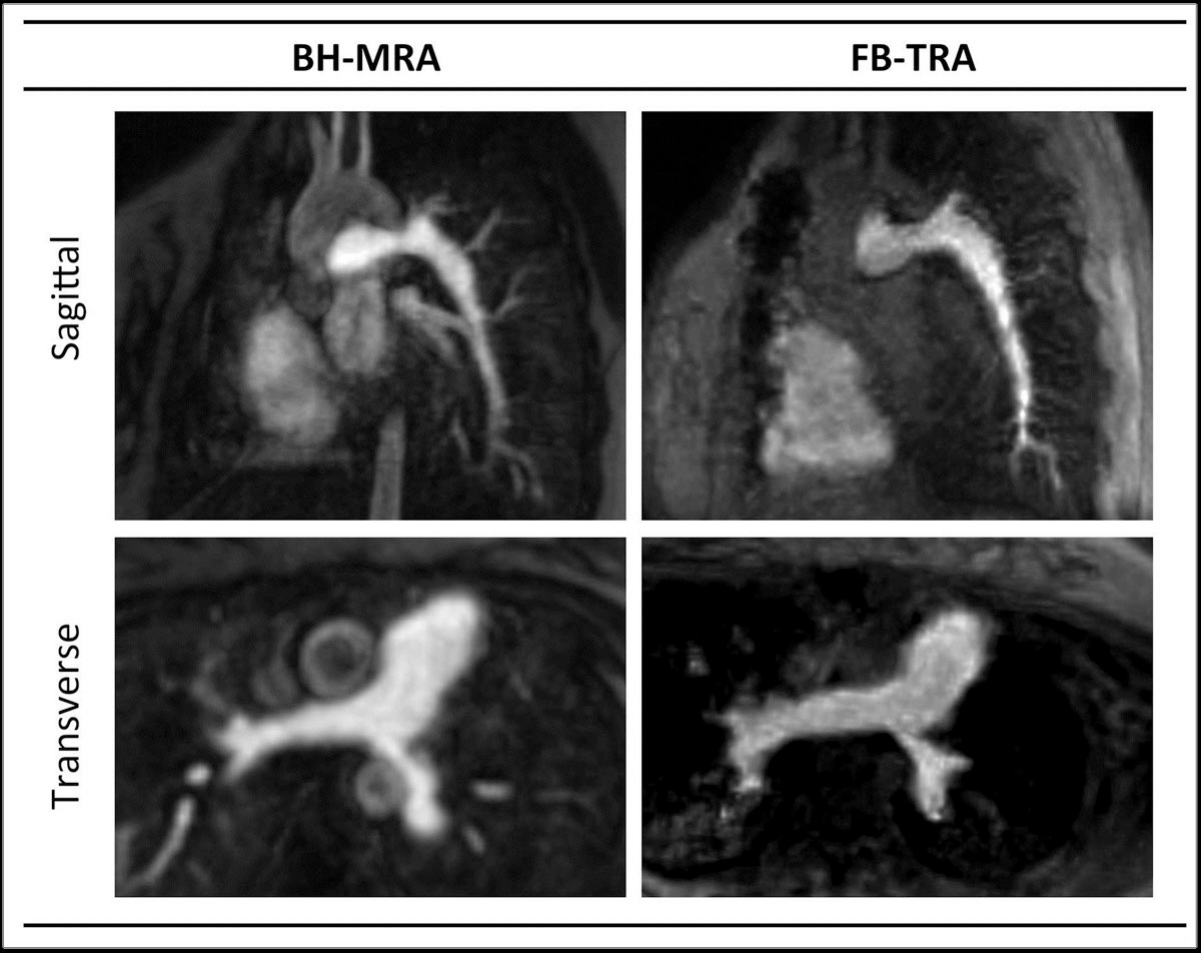


Example images are shown in figure 1. BH-MRA had significantly higher SNR (P<0.0001), CNR (P<0.0001) and RC (P=0.02) compared to the FB-TRA images. However, edge sharpness was significantly (P<0.0001) higher in FB-TRA compared to BH-MRA.

## Conclusions

We have described a FB-TRA technique that has been shown to enable accurate diagnosis and vessel measures compared to conventional BH-MRA. This technique simplifies the MRA technique by eliminating the need for bolus timing, and will enable angiography to be performed on children and adults whom find breath-holding difficult.

## Funding

N/A.

